# Response to comment on: Could internal limiting membrane peeling before Voretigen Neparvovec-ryzl subretinal injection prevent focal chorioretinal atrophy?

**DOI:** 10.1016/j.heliyon.2024.e37945

**Published:** 2024-09-19

**Authors:** Lea Dormegny, Fouzia Studer, Arnaud Sauer, Laurent Ballonzoli, Claude Speeg-Schatz, Tristan Bourcier, Helene Dollfus, David Gaucher

**Affiliations:** Department of Ophthalmology, New Civil Hospital, Strasbourg University Hospitals, Strasbourg, France; Institut de Génétique Médicale d'Alsace, CARGO Reference Center for Rare Diseases in Genetic Ophthalmology, University Hospitals of Strasbourg, Strasbourg, France; Department of Ophthalmology, New Civil Hospital, Strasbourg University Hospitals, Strasbourg, France; Institut de Génétique Médicale d'Alsace, CARGO Reference Center for Rare Diseases in Genetic Ophthalmology, University Hospitals of Strasbourg, Strasbourg, France; Department of Ophthalmology, New Civil Hospital, Strasbourg University Hospitals, Strasbourg, France

We read with great interest the comments of Bjelos and associates on our work entitled “Could internal limiting membrane peeling before Voretigen Neparvovec-ryzl subretinal injection prevent focal chorioretinal atrophy?”.

The authors first asked for further information on the peri-operative immunomodulatory therapy administered to the patients (dosage, tolerance and period). They asked if the treatment was initiated prior to the surgery and if chorioretinal atrophy at the injection site developed in the first or the second injected eye. They questioned the role of the immunosuppressant on post operative outcomes and hypothesized that the stability of immunosuppressants in the bloodstream might have affected the incidence of atrophy in the second injected eye.

For the three patients presented here, the immunomodulatory therapy was initiated prior to the operation, as recommended by the Summary of the Product Characteristics (SmPC). Prednisolone (two tablets of 20mg) was prescribed three days prior to the intervention and continued until four days after. Dosage was then progressively reduced for 10 days. Additional topic steroids and non-steroids anti-inflammatory treatment was administrated for 3 weeks in the operated eye. In the two patients presenting with unilateral chorioretinal atrophy at the injection site, atrophy developed in the first injected eye, while the second remained free of local atrophy. Injections were performed with an interval of 19 days. Although in these two patients, immunomodulatory treatment was continued between the two eyes, the third patient remained free of local atrophy in both eyes. In this patient, focal ILM peeling was performed in both eyes and injections were performed with an interval of 21 days.

Although our series only reported the outcomes of three patients, other patients have received Voretigen Neparvovec-ryzl subretinal injection since our results were published. A 22-year-old patient received Voretigen Neparvovec-ryzl subretinal injection after focal ILM peeling on his right eye. He did not take the immunomodulatory treatment as recommended, nor before nor after the injection. However, he did not develop chorioretinal atrophy at the injection site after one year of follow up (see Fig. 1 below: ultrawide field retinography before (A) and one year after the injection (B)). A 4-year-old girl received Voretigen Neparvovec-ryzl subretinal injection on her left eye after focal ILM peeling. She took the oral corticotherapy as recommended by the SmPC and did not develop chorioretinal atrophy at the injection site after 6 months of follow up (see Fig. 2 below: ultrawide field retinography before (A) and one year after the injection (B)).

**Fig. 1:**
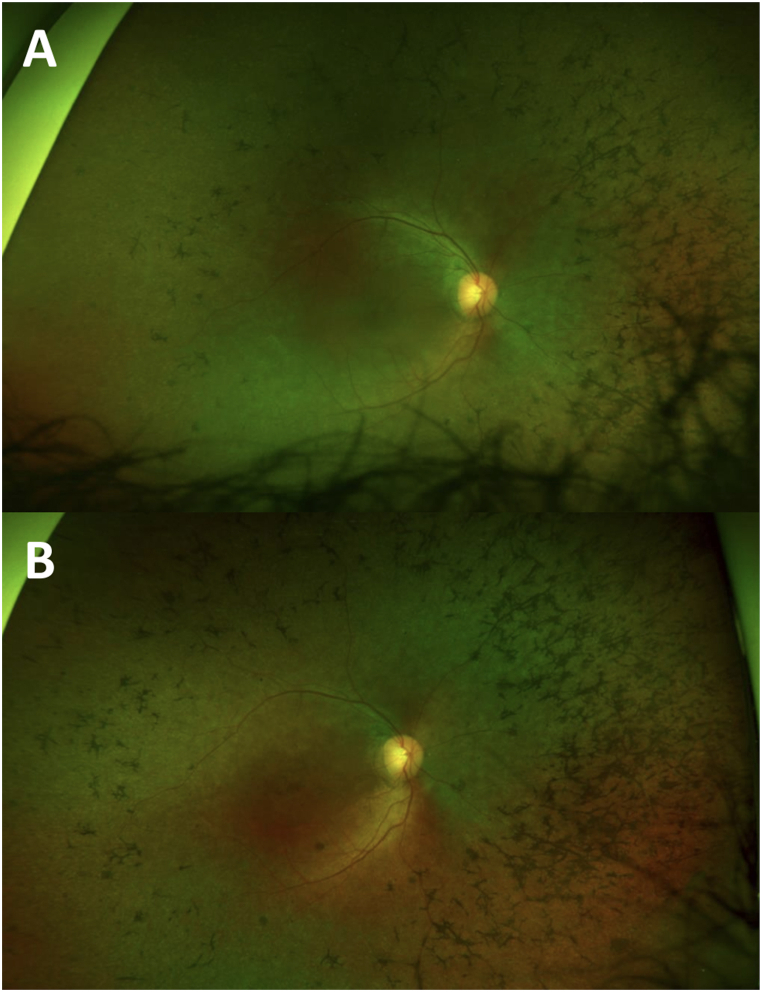
Ultrawide field retinography of the right eye of a 22-year-old boy who received Voretigen Neparvovec-ryzl subretinal injection after ILM peeling at the injection site: preoperative fundus (A) and one-year postoperative fundus (B).

**Fig. 2:**
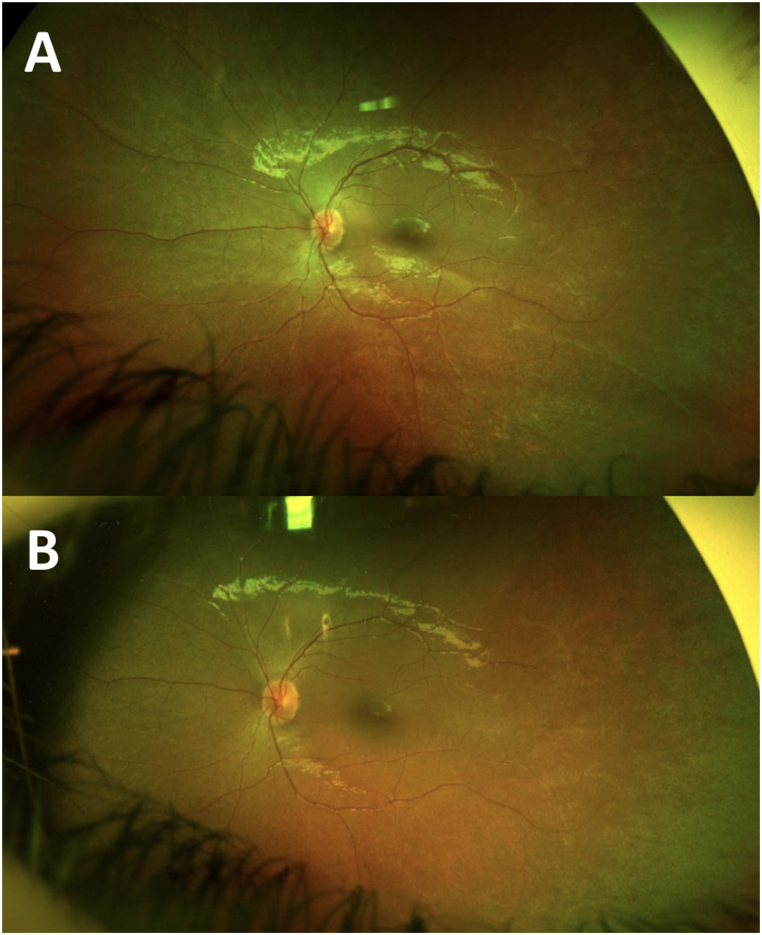
Ultrawide field retinography of the left eye of a 4-year-old girl who received Voretigen Neparvovec-ryzl subretinal injection after ILM peeling at the injection site: preoperative fundus (A) and six months postoperative fundus (B).

In conclusion, we believe that immune system response can lead to retinal atrophy after Voretigen Neparvovec-ryzl treatment, and it is legitimate to wonder if the duration of the treatment with steroids initiated for the first eye until the intervention of the second eye may influence the occurrence of such complication at the injection site. Our series is too small to conclude with certainty on this hypothesis. However, the fact that no atrophy occurred in any of the treated eye with focal ILM peeling, even when steroid treatment was recently administered or not administered, is not in favor of the latter hypothesis. We strongly believe that surgical technique is greatly related to the occurrence of focal chorioretinal atrophy at the injection site after Voretigen Neparvovec-ryzl injection.

## CRediT authorship contribution statement

**Lea Dormegny:** Data curation, Formal analysis, Investigation, Writing – original draft. **Fouzia Studer:** Data curation, Methodology, Resources, Writing – original draft. **Arnaud Sauer:** Formal analysis, Methodology, Supervision, Writing – review & editing. **Laurent Ballonzoli:** Validation, Visualization, Writing – original draft. **Claude Speeg-Schatz:** Data curation, Resources, Visualization, Writing – review & editing. **Tristan Bourcier:** Formal analysis, Supervision, Visualization, Writing – review & editing. **Helene Dollfus:** Conceptualization, Resources, Supervision, Validation, Writing – review & editing. **David Gaucher:** Conceptualization, Methodology, Supervision, Writing – review & editing.

## Declaration of competing interest

The authors declare that they have no known competing financial interests or personal relationships that could have appeared to influence the work reported in this paper.

